# Supplementary material for: Delineation of Grade II and III Gliomas Investigated by 7T MRI: An Inter-Observer Pilot Study
Source: Diagnostics (Basel). 2023 Apr 7;13(8):1365. doi: 10.3390/diagnostics13081365 (PMC10137409; doi:10.3390/diagnostics13081365)
Supplement: Supplementary file 1 [file diagnostics-13-01365-s001.zip › diagnostics-2304059-supplementary.pdf]

Supplementary Material

Supplementary Table S1. Relative GTV size from 3T compared to 7T

	N patients per category						
**T2 Observer 1**	All (7)	Big tumor (4)	Small tumor (3)*	Grade II (4) (Ast. + olig.)	Grade III (3) (Ast. + olig.)	Astrocytoma (5)	Oligodendroglioma (1)
(7T < 3T)	6	4	2	4	2	4	1
(7T = 3T)	1	0	1	0	1	1	0
(7T > 3T)	0	0	0	0	0	0	0
**T2 Observer 2**							
(7T < 3T)	4	2	2	2	2	2	1
(7T = 3T)	1	1	0	1	0	1	0
(7T > 3T)	2	1	1	1	1	2	0
**FLAIR Observer 1**							
(7T < 3T)	3	3	0	1	1	3	0
(7T = 3T)	4	1	3	3	2	2	1
(7T > 3T)	0	0	0	0	0	0	0
**FLAIR Observer 2**							
(7T < 3T)	5	2	3	3	2	3	1
(7T = 3T)	1	1	0	1	0	1	0
(7T > 3T)	1	1	0	0	1	1	0

Same size is defined as a difference of less than ±5%. Big tumor was defined as above 15 cm^3. One astrocytoma patient had both the astrocytoma and oligodendroglioma characteristics.
*The patient without histology verification is in the grading category as a grade 2 based on the MR findings.
